# Thanatometabolomics: introducing NMR-based metabolomics to identify metabolic biomarkers of the time of death

**DOI:** 10.1007/s11306-019-1498-1

**Published:** 2019-03-05

**Authors:** Marina Mora-Ortiz, Marianne Trichard, Alain Oregioni, Sandrine P. Claus

**Affiliations:** 1 Department of Food and Nutritional Sciences, The University of Reading, Whiteknights Campus, Reading, RG6 6AP UK; 2 Department of Twin Research, Kings College London, St Thomas’ Hospital Campus, 3rd Floor South Wing Block D, Westminster Bridge Road, London, SE1 7EH UK; 3 Département Biologie Alimentaire à l’Ecole Nationale Supérieure de Chimie, Biologie et Physique de Bordeaux (ENSCBP), 33600 Pessac, France; 4 MRC Biomedical NMR Centre, The Francis Crick Institute, 1 Midland Road, London, NW1 1AT UK

**Keywords:** Post-mortem metabolome, Nuclear magnetic resonance spectroscopy (NMR), Biomarkers, Forensic research

## Abstract

**Introduction:**

Death is the permanent cessation of the critical functions of the organism as a whole. However, the shutdown of a complex biological organism does not abruptly terminate at time of death. New high-throughput technologies allow the systematic investigation of the biochemical modulations occurring after death. Recent genomics studies have demonstrated that genes remain active after death, triggering upregulation of some genes and initiating feedback loops. These genes were mostly involved in pathways related to immunity, inflammation and cancer. These genetic modulations suggest many biochemical events persist after death, which can be captured using a metabolomics approach.

**Objectives:**

This proof of concept work aimed to determine whether NMR spectroscopy could identify metabolomics changes occurring after death, and characterise the nature of these metabolomics modulations.

**Methods:**

High-resolution ^1^H-NMR spectroscopy was applied to six biological matrices: heart, kidney, liver, spleen, skin and white adipose tissue of ten adult mice at three different type points.

**Results:**

Forty-three metabolites were associated with post mortem metabolomics modulations. Kidney, heart and spleen showed the highest metabolic perturbations. Conversely, skin and white adipose tissue were the least altered matrices. Early metabolic modulations were associated with energy metabolism and DNA synthesis, by contrast, late metabolomics modulations were associated with microbial metabolism.

**Conclusions:**

NMR has proven potential to determine the time of death based on post-mortem metabolomics modulations. This could be useful in the context of transplants, forensic studies and as internal quality control in metabolomics studies. Further investigations are required to validate these findings in humans in order to determine which compounds robustly reflect post-mortem metabolic fluctuations to accurately determine the time of death.

**Electronic supplementary material:**

The online version of this article (10.1007/s11306-019-1498-1) contains supplementary material, which is available to authorized users.

## Introduction

The definition of death, the end of the life of an organism, has evolved along history as technology has progressed, from a simple cardiorespiratory centred concept to a modern neurocentric diagnosis (Laureys 2005). Nowadays, the most accepted description of death is “permanent cessation of the critical functions of the organism as a whole” (Bernat 1998). However, not all the functions cease simultaneously, and the shutdown of a complex biological organism, such as a human being, does not abruptly terminate at time of death (Pozhitkov et al. 2016).

The shutting down of an individual, consequent disassembly and biochemical pathways involved, have been little investigated so far, but the advent of new high-throughput technologies brings in new possibilities in the area of genetic, epigenetic, proteomic and metabolomics. A better understanding of biochemical events occurring after death is critical to maximize the success of organ transplantation, cancer research and may contribute to criminal investigations (Mitch 2016).

During a forensic investigation, the estimation of the post-mortem interval (PMI) is normally based on factors such as the body’s core temperature, livor mortis, rigor mortis or other evidence external to the body itself, such as the time when the last registered phone activity occurred. In order to accurately evaluate the time of death, various chemical methods have been established since the early 1970s’ using samples from liver, skeletal muscle, vitreous humour, cerebrospinal fluid and serum. However, the precise estimation of the PMI remains a challenge (Mitch 2016; Ith et al. 2002; Coe 1974; Endo et al. 1990; Muñoz et al. 2001; Bocaz-Beneventi et al. 2002; Mittmeyer and Welte 1982; Bonte 1975).

Preliminary genetic studies have shown in deceased humans, mice and zebrafish models, that some genes remain active up to four days after death. Not only were these genes active, but they also upregulated or initiated feedback loops including upregulation of immunity, inflammation and cancer genes within one hour from death. It has also been hypothesized by Pozhitkov and colleagues (2016) that immune reaction against some transplanted organs could be linked to the genetic activity of the organ itself, rather than to immunosuppressive agents (Aberg et al. 2008; Haagsma et al. 2001). It was suggested that the risk of suffering a cancer post organ transplantation is associated with pro- oncogenic genes expressed after death (Burra and Rodriguez-Castro 2015; Pozhitkov et al. 2016; Schrem et al. 2016). Increase in transcript abundance after death was attributed to thermodynamics and kinetic dynamics including epigenetic changes (Pozhitkov et al. 2016). These processes have been hypothesised to be linked with changes in the wrapping of the nucleosome, nucleopores and ion/solute protein channels (Mattson and Chan 2003; Lang et al. 2005; Pozhitkov et al. 2016).

Consistent with Pozhitkov and colleagues’s findings, previous studies in human cadavers, using reverse transcription real-time quantitative PCR (RT-RT qPCR), also showed active post-mortem activity resulting in gene upregulation twelve hours after death affecting myosin light chain 3 (*Myl3*), vascular endothelial growth factor A (*Vegfa*) and matrix metalloprotease 9 (*Mmp9*) genes (González-Herrera et al. 2013). This suggests that significant biochemical modulations occur after death, which may be informative to determine PMI.

Metabolomics is an established method to study the metabolome of an organism using high-resolution untargeted spectroscopy approaches such as nuclear magnetic resonance (NMR) spectroscopy and mass spectrometry (MS) (Daviss 2005; Nicholson 2006; Nicholson et al. 1999; Klug et al. 2012; Bernot 2004; Blackstock and Weir 1999). A similar approach has been applied to study post-mortem samples of brain from sheep and selected human samples (Ith et al. 2002) using ^1^H magnetic resonance spectroscopy (^1^H-MRS), and the rat retina by gas chromatography-mass spectrometry (GC-MS) and ultra high performance liquid chromatography-mass spectrometry (UHPLC-MS) (Tan et al. 2016). ^1^H-NMR has been applied to identify post-mortem changes in food appropriately preserved for human consumption such as salmon, chicken and beef (Shumilina et al. 2015; Schreurs 2000; Graham et al. 2010).

Many animals such as pigs, humans, rodents, chickens, and horse have been metabolically characterised by ^1^H NMR spectroscopy in the recent past (Claus et al. 2008; Le Roy et al. 2016; Martin et al. 2007; Merrifield et al. 2011; Ndagijimana et al. 2009; Holmes et al. 1997; Escalona et al. 2015), but to the best of our knowledge, there is no exhaustive metabolomics ^1^H NMR-based characterisation of post-mortem changes.

A better understanding of molecular changes occurring after death would help in transplantation and cancer research, forensic sciences and as quality control for metabolomics analyses. Thus, the aim of this study was to characterise which metabolomics changes occur after death, and whether or not NMR spectroscopy could identify metabolomics modulations between three post-mortem time points in different tissues from a common rodent model.

## Materials and methods

### Animal design

Ten 4-week-old C57BLKS *db*/+ mice (females, n = 6; males, n = 4) were acquired from Taconic Bioscience. Bedding was mixed between cages to minimise cage effect due to variations in the microbial environment on a weekly basis. Mice were humanly euthanized by neck dislocation, according to the specifications of the United Kingdom Animals Scientific Procedures Act, 1986 (ASPA) when they were one year old. None of these animals displayed any sign of illness and they were assumed to be healthy at the time of death. Animal procedures were conducted according to the UK ethical legislation on animal experimentation (ASPA 1986) and approved by the Home Office (PPL 70/7942).

Bodies were kept at room temperature until time of dissection immediately after euthanasia (Time Point 1, n = 3), six hours after euthanasia (Time Point 2, n = 3) and 24 h after euthanasia (Time Point 3, n = 4).

Heart, kidney, liver, spleen, skin and White Adipose Tissue (WAT) were collected and immediately frozen in liquid nitrogen and kept at − 80 °C until analysis.

### Sample preparation

Tissue biopsies (~ 70 mg) were cut on a frozen surface to prevent metabolic degradation. Samples were homogenised into closed sterile microtubes with 1 mL methanol/water (1:1) using a Tissue Lyser LT (Qiagen, Germany) and 1 mm diameter zirconia beads. In the case of WAT samples, 0.5 mL of methanol/water (3:1) added to 0.3 mL of Chloroform were used to separate lipids from the polar phase. The homogenate was then centrifuged at 5000×*g* for 5 min at 4 °C. The supernatants of these centrifugation products were evaporated in a speed vacuum concentrator (Eppendorf, Germany) at 30 °C for 4 h. These polar extracts were reconstituted in 200 µL of phosphate buffer and transferred into 3 mm NMR tubes with code. Samples were kept in a SampleJet (Bruker Biopsin, Rheinstetten, Germany) at 5.8 ± 0.1 °C until ^1^H NMR acquisition at 25 °C. The acquisition time per sample was 15 min.

### NMR analysis

A Bruker Avance HD 700 MHz (Bruker Biopsin, Rheinstetten, Germany) equipped with a cooled SampleJet and a TCI CryoProbe from the same manufacturer was used to acquire the spectra from all biofluids. The data was recorded at 25 °C ± 0.15 °C calibrated with a Methanol-D4 sample (Findeisen et al. 2007).

One-dimensional ^1^H NMR-based experiment, Carr-Purcell-Meiboom-Gill (CPMG) (Meiboom and Gill 1958), was recorded with presaturation applied for 3 s during the relaxation delay at a B_1_ field of 50 Hz, 64 scans, 8 dummy scans and 32 k complex pairs over a spectral width of 9091 Hz. CPMG T_2_ filter was set at 39 ms (122 echoes of 316 µs).

### Data processing and statistical analysis

MestReNova version 11.0.2-18153 (Mestrelab Research S.L., Spain) was used to pre-process spectra. Manual phase correction and automatic baseline correction were performed using the Whittaker Smoother algorithm. Calibrations were carried out using TSP (δ 0.00 ppm). Residual water (δ 4.70 ppm − 5.10 ppm) and noise (regions before δ 0.5 ppm and after δ 9.5 ppm) were removed before exporting spectra for statistical analysis.

Matlab version R2015b (Mathworks, UK) was used to carry out the statistical analysis using algorithms provided by the Korrigan Toolbox version 0.1 (Korrigan Sciences Ltd., U.K.). After normalisation using a median-base probabilistic quotient method (Dieterle et al. 2006) sample variability was evaluated using an unsupervised Principal Component Analysis (PCA). Supervised pairwise Orthogonal Projection to Latent Structures Discriminant Analysis (O-PLS DA) was used to identify metabolomics modulations associated with the time of death. Seven-fold cross-validation was used to evaluate the goodness of prediction (*Q*^2^Y value) of the O-PLS DA models. The scores are shown as a plot of the model scores (T) on the x axis against the cross-validated scores (Tcv) on the y axis. This allows to visualise the level of overfit since overfitted models would scatter away from the diagonal. The model loadings plot was colour-coded to display metabolites that are strongly associated with the discriminant component in warm colours (colours close to red); by contrast, metabolites in cold colours (colours close to blue) are not discriminant.

Chenomx NMR Suite 8.2 from Chenomx Inc (Edmonton, Canada), The Human Metabolome Database (http://www.hmdb.ca) and available literature (Claus et al. 2011, 2008; de Castro et al. 2013; Le Roy et al. 2016) were used for metabolite identification. This pipeline is a standard method in the field and has been previously employed by our group and other groups (Castro et al. 2013; Le Roy et al. 2016; Claus et al. 2011, 2008; Giallourou et al. 2018) as being considered an acceptable NMR data analysis approach.

## Results

Determination of time of death still represent a challenge for the scientific community. In this investigation, we combined NMR metabolic profiling with multivariate statistics to evaluate sequential metabolic modulations after death. Principal Component Analyses (PCA) were carried out on all datasets to evaluate sample variability. Specific metabolic modulations were more accurately evaluated using pairwise supervised O-PLS DA analyses comparing Time Point 1 (immediately after euthanasia), Time Point 2 (six hours after euthanasia) and Time Point 3 (twenty-four hours after euthanasia).

**Fig. 1:**
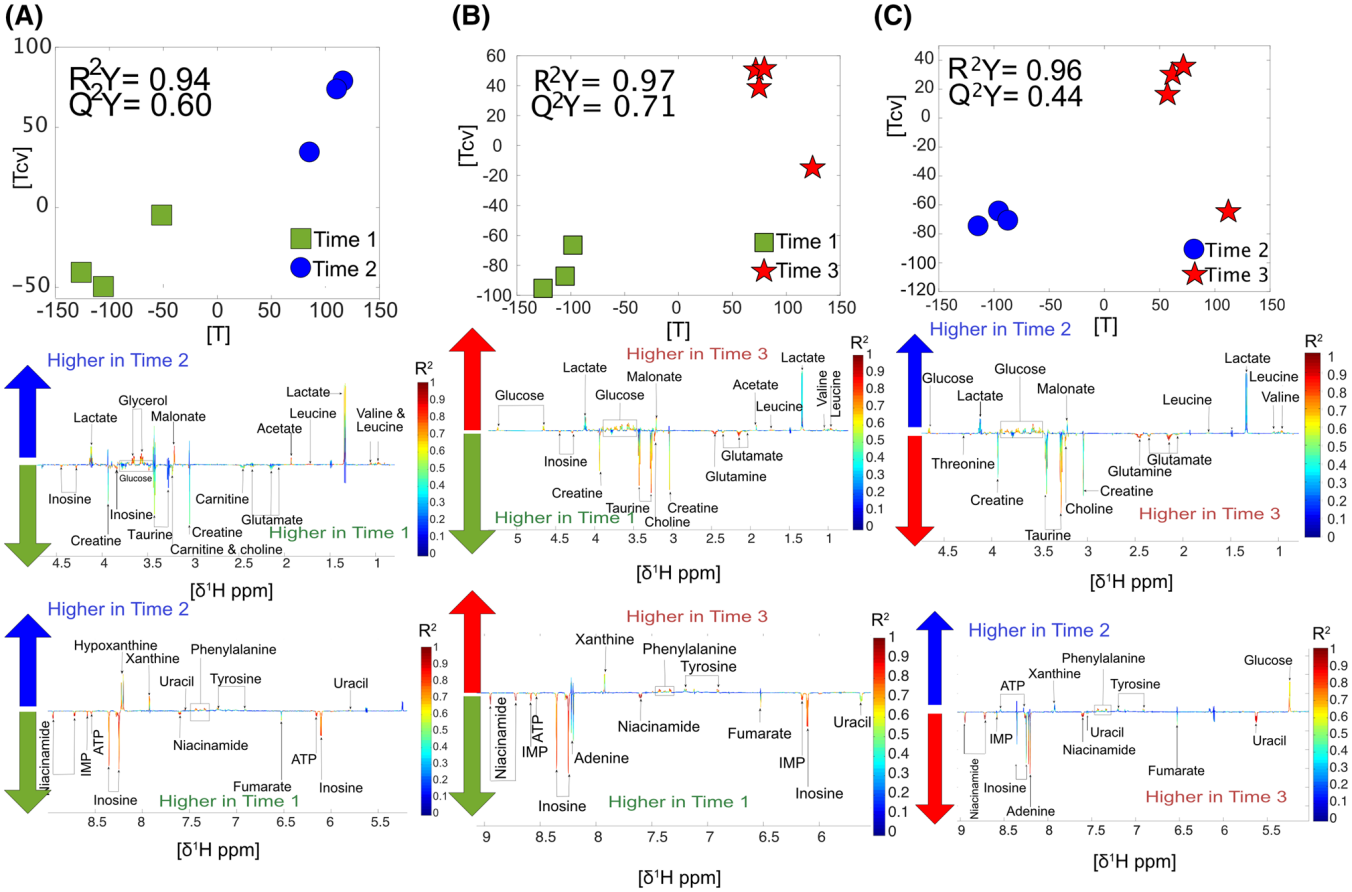
Heart: **a** Time point 1 versus time point 2. Score and loading plots from the O-PLS DA model (n = 6). **b** Time point 1 versus time point 3. Score and loading and plot from the O-PLS DA model (n = 7). **c** Time point 2 versus time point 3. Score and loading plots the O-PLS DA (n = 7)

*Heart* was one of the tissues that underwent the most metabolic modulations after death. In total, 65 metabolic modulations occurred when comparing the three-different time points. These modulations were evenly distributed between the three possible pairwise comparisons. In the comparison between TP1 versus TP2 (n = 6, R^2^Y = 0.94, Q^2^Y = 0.60, Fig. 1a) inosine and ATP were the two metabolites that were drastically decreased in TP2 compared to TP1; by contrast, glycerol and malonate were higher in TP2 than in TP1. TP3 compared to TP1 (n = 7, R^2^Y = 0.97, Q^2^Y = 0.71, Fig. 1b) had significantly higher levels of phenylalanine and some branched chain amino acids. Conversely, TP3 had lower levels of niacinamide, energy transporters such as IMP and ATP, and inosine. Finally, the comparison between TP2 and TP3 (n = 7, R^2^Y = 0.96, Q^2^Y = 0.44, Fig. 1c) showed that the main differences driving the model came from the increase of adenine and niacinamide in TP3. Other metabolites fluctuating between these time points are reported in Fig. 1 and Supplementary material S1.

**Fig. 2:**
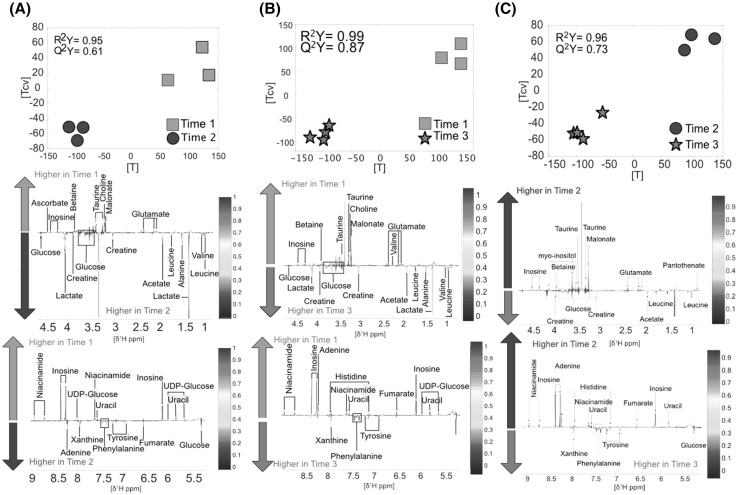
Kidney: **a** Time point 1 versus time point 2. Score and loading plots from the O-PLS DA model (n = 6). **b** Time point 1 versus time point 3. Score and loading and plot from the O-PLS DA model (n = 7). **c** Time point 2 versus time point 3. Score and loading plots the O-PLS DA (n = 7)

*Kidney* was another tissue showing numerous metabolic alterations after death reflected by a total of 70 metabolic fluctuations. Metabolomics differences occurring in kidney after death principally included lower levels of inosine and higher levels of lactate in TP2 compared to TP1 (n = 6, R^2^Y = 0.95, Q^2^Y = 0.61, Fig. 2a). In the model comparing TP1 and TP3 (n = 7, R^2^Y = 0.99, Q^2^Y = 0.87, Fig. 2b), differences were due to significantly lower levels of inosine, uracil and taurine. TP3 by contrast had higher levels of phenylalanine and tyrosine. Accordingly, metabolic differences observed in kidney tissue between TP2 and TP3 included lower levels of inosine, niacinamide and adenine and significantly higher levels of phenylalanine and tyrosine in TP3. More details about metabolic fluctuations between different time points are provided in Fig. 2 and Supplementary material S1.

**Fig. 3:**
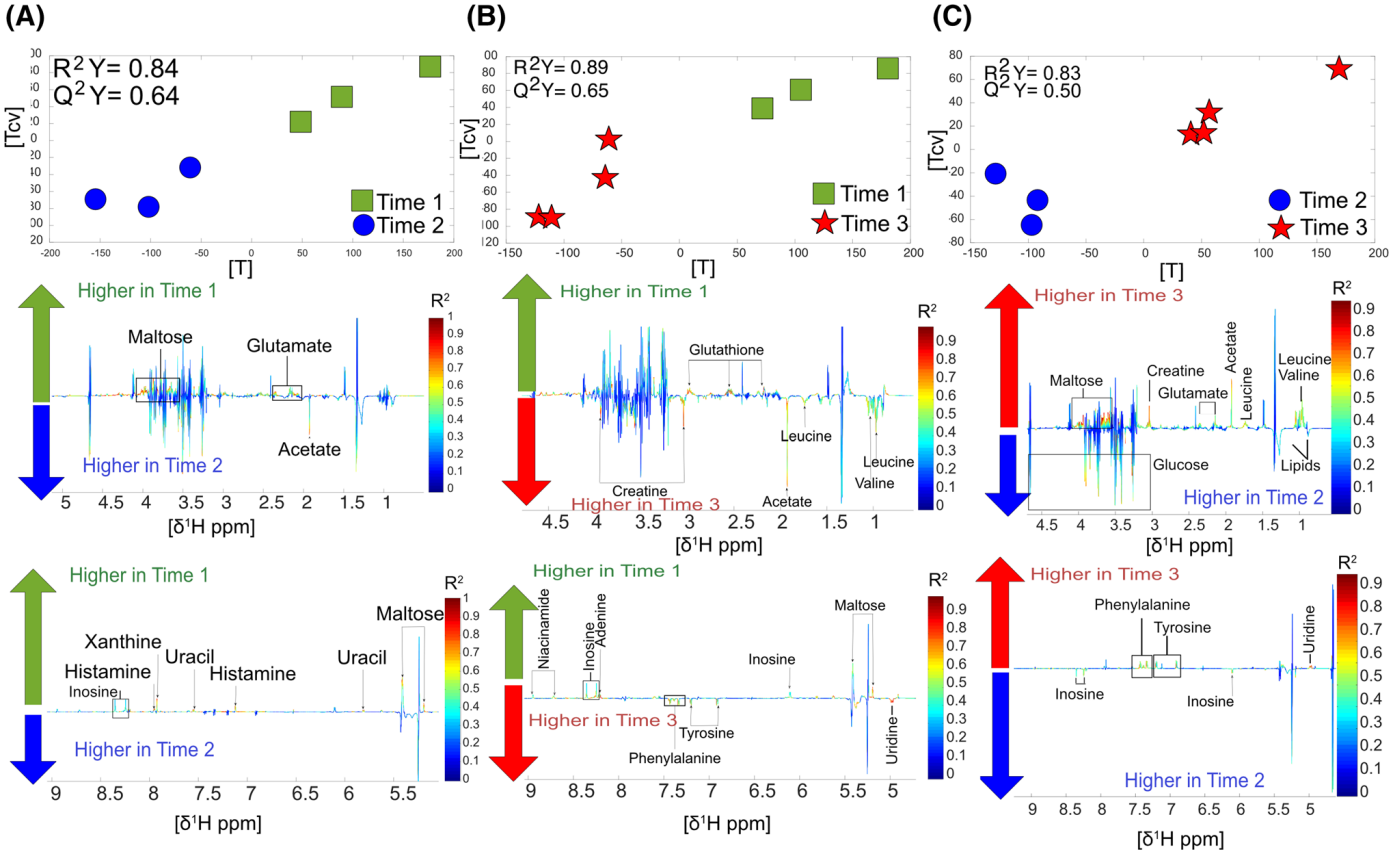
Liver: **a** Time point 1 versus time point 2. Score and loading plots from the O-PLS DA model (n = 6). **b** Time point 1 versus time point 3. Score and loading and plot from the O-PLS DA model (n = 7). **c** Time point 2 versus time point 3. Score and loading plots the O-PLS DA (n = 7)

The *liver*, unlike the previous organs, displayed only 34 metabolic differences in total, which indicates that this organ has some resistance to metabolic degradation after death. Differences between TP1 and TP2 were mainly characterised by a decrease of maltose and glutamate in TP2 compared to TP1, and an increase of acetate which is a sign of degradation for acetyl-coA which cannot enter the Krebs cycle in the mitochondria anymore due to lack of oxygen (n = 6, R^2^Y = 0.84, Q^2^Y = 0.64, Fig. 3a). TP3 showed significantly higher levels of creatine compared to TP1, while glutathione levels were drastically decreased (n = 7, R^2^Y = 0.89, Q^2^Y = 0.65, Fig. 3b). Finally, TP3 also showed higher levels of maltose and creatine, and had slightly lower levels of glucose than TP2 (n = 7, R^2^Y = 0.83, Q^2^Y = 0.50, Fig. 3c).

**Fig. 4:**
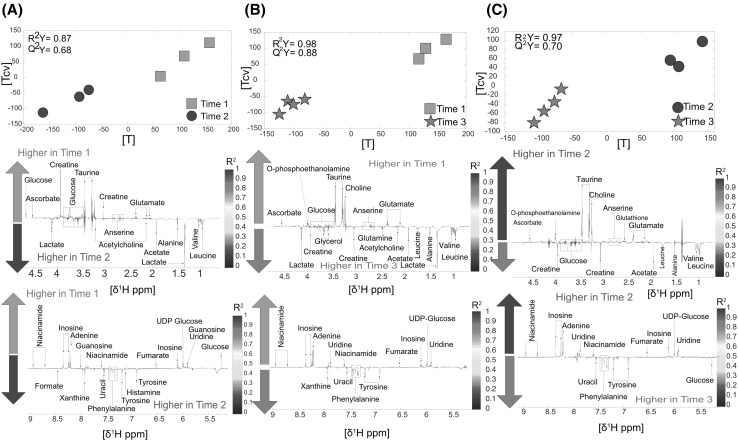
Spleen: **a** Time point 1 versus time point 2. Score and loading plots from the O-PLS DA model (n = 6). **b** Time point 1 versus time point 3. Score and loading and plot from the O-PLS DA model (n = 7). **c** Time point 2 versus time point 3. Score and loading plots the O-PLS DA (n = 7)

The organ showing the highest number of metabolic fluctuations was *spleen*, that counted for 75 modulations in total between different time points. During the 6 h of lapse time between TP1 and TP2, lactate and acetate increased. Conversely, glucose, creatine and taurine significantly decreased six hours after death (n = 6, R^2^Y = 0.87, Q^2^Y = 0.68, Fig. 4a). When comparing TP1 and TP3, taurine, choline, inosine and adenine were significantly lower at TP3, while phenylalanine and tyrosine increased (n = 7, R^2^Y = 0.98, Q^2^Y = 0.88, Fig. 4b). Finally, the metabolic differences between TP2 and 3 were represented by lower levels of taurine, choline and glutamate in TP3, and higher levels of creatine and phenylalanine (n = 7, R^2^Y = 0.97, Q^2^Y = 0.70, Fig. 4c).

**Fig. 5:**
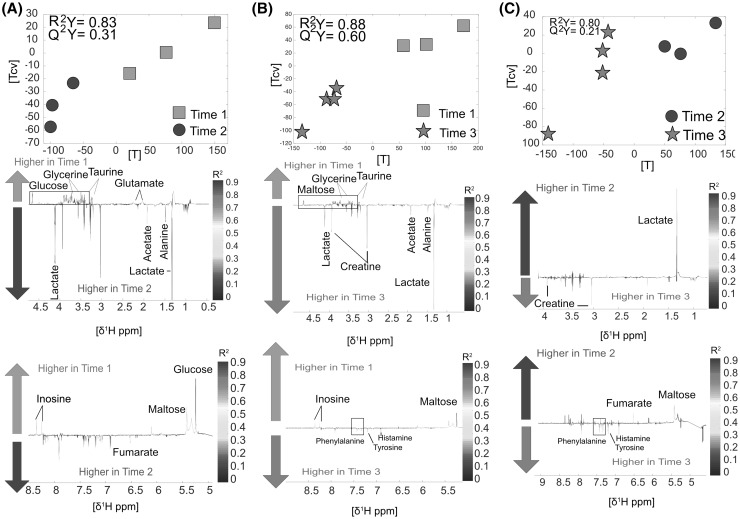
Skin **a** Time point 1 versus time point 2. Score and loading plots from the O-PLS DA model (n = 6). **b** Time point 1 versus time point 3. Score and loading and plot from the O-PLS DA model (n = 7). **c** Time point 2 versus time point 3. Score and loading plots the O-PLS DA (n = 7)

*Skin* samples showed little differences over time, counting only twenty-eight detectable metabolic modulations in total. These differences included for example higher levels of lactate after 6 h from the time of death (TP2), which could be associated with bacterial activity, and lower levels of glucose and taurine (n = 6, R^2^Y = 0.83, Q^2^Y = 0.31, Fig. 5a). Differences between TP1 and TP3 were similar to the metabolic variations observed between TP1 and TP2, besides increased levels of creatine, phenylalanine, tyrosine and histamine (n = 7, R^2^Y = 0.88, Q^2^Y = 0.60, Fig. 5b) at TP3. The metabolic comparison between TP2 and TP3 showed lower levels of lactate, fumarate and maltose and higher levels of phenylalanine, tyrosine and histamine at TP3 (n = 7, R^2^Y = 0.80, Q^2^Y = 0.21, Fig. 5c). All the metabolic fluctuations are summarised in the Supplementary material S1.

**Fig. 6:**
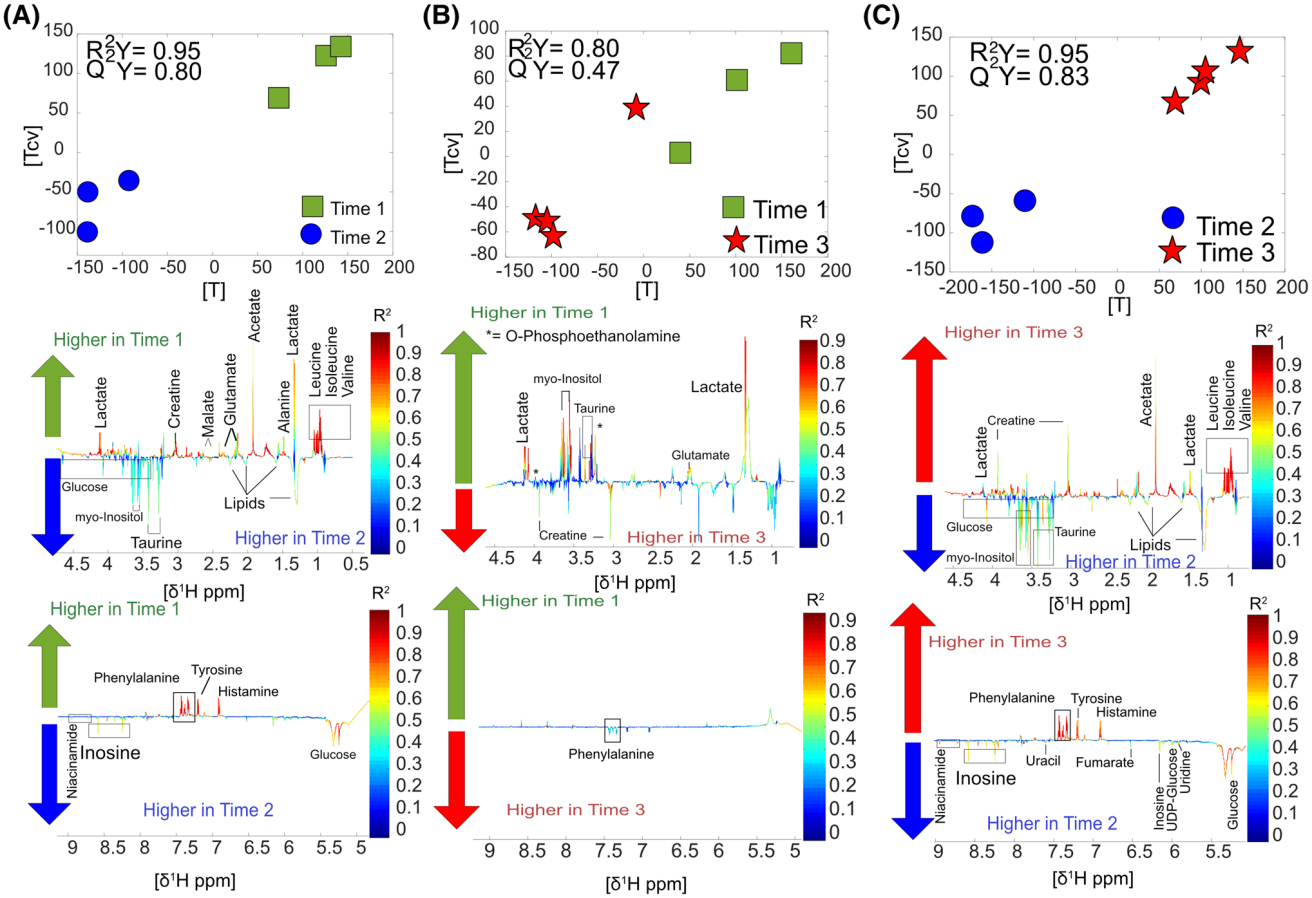
WAT: **a** Time point 1 versus time point 2. Score and loading plots from the O-PLS DA model (n = 6). **b** Time point 1 versus time point 3. Score and loading and plot from the O-PLS DA model (n = 7). **c** Time point 2 versus time point 3. Score and loading plots the O-PLS DA (n = 7)

The metabolomics analysis of the polar phase of *WAT* showed that some metabolites including leucine, isoleucine and lactate were lower at TP2 compared to TP1; however, there were no significant metabolic increases after six hours (TP2) (n = 6, R^2^Y = 0.95, Q^2^Y = 0.80, Fig. 6a). TP3 compared to TP1 had lower levels of lactate, *myo*-inositol and taurine; similarly to the previous model, no significant metabolic increases were observed (n = 7, R^2^Y = 0.80, Q^2^Y = 0.47, Fig. 6b). Some metabolic fluctuations were observed between TP3 and TP2, where the former had clearly higher levels of lactate and branched chain amino acids (n = 7, R^2^Y = 0.95, Q^2^Y = 0.83, Fig. 6c). More details about metabolic fluctuations are provided in Supplementary material S1.

## Discussion

^1^H NMR-based metabolomics fingerprinting has been previously used to characterised the metabolome of pigs, humans, rodent, chicken, horses and even the metabolomics changes occurring in diseases such as Type 2 Diabetes in a rodent model (Claus et al. 2008; Le Roy et al. 2016; Martin et al. 2007; Merrifield et al. 2011; Ndagijimana et al. 2009; Holmes et al. 1997; Escalona et al. 2015; Mora-Ortiz et al. 2018). However, to date, little metabolomic approaches have been used to characterise PM biochemical modulations. Previously, PM analysis were based on temperature, the golden standard being the Henssge nomogram method (based on the two-exponential model of body cooling). Other complementary methods are the compound method, which uses the skeletal muscle electrical and mechanical excitability, post-mortem lividity, rigor mortis and pharmacological excitability of the iris (Medea 2016).

The present study is a proof of concept work that aimed to determine whether NMR-based metabolomics has potential to distinguish biochemical changes at different time points after death. Results show that 43 metabolites have been linked to PM changes overtime, the kidney and spleen being the two organs that showed the highest modulations, and skin the organ showing the least metabolic fluctuations. This may be due to oxygen permeation through the skin (Stücker et al. 2002), which may delay the activation of anaerobic pathways in this tissue. Some of the metabolites that were more frequently associated with PM metabolic fluctuations included taurine, niacinamide, phenylalanine, tyrosine, glycerol, xanthine and lactate.

Lactate was a metabolite often found modulated in different matrices. When little amounts of oxygen are available, the anaerobic glycolysis of glucose reserves directs pyruvate to lactate production instead of entering the Krebs cycle under normal aerobic conditions (Supplementary material S2). As a consequence, lactate is known to accumulate in anaerobic conditions (Simchowitz and Textor 1992; Chen et al. 2017). In mammals, lactate can be recycled into glucose in the liver, which is likely the reason why this organ does not show fluctuations in this metabolite unlike heart, kidney and spleen for example (Cox and Nelson 2008). To the contrary, WAT metabolic profiles contained lower levels of lactate in TP2 and TP3 than in TP1. The WAT is an important contributor to circulating lactate levels (DiGirolamo et al. 1992). Therefore, decreased lactate levels in the WAT may be resulting from the shuttle of lactate towards remote organs during anoxia (Brooks 2009; Gladden 2004). This lactate shuttle has been associated with gene expression involved in lactate removal (Gladden 2008; Hashimoto and Brooks 2008; Brooks 2009), which means that in a PM context lactate may support gene transcription as previously described by Pozhitkov and colleagues (2016). Other metabolites derived from energy metabolism pathways, like glycerol that results mostly from lipolysis (Nielsen et al. 2014), were modulated in organs such as the spleen.

The purine degradation pathway produced several metabolites (IMP, Inosine, Hypoxanthine and Xanthine) identified in various organs such as the liver, heart and spleen (Al-Khalidi and Chaglassian 1965). Since end-product metabolites from this pathway, such as allantoin, were not modulated, further nucleic acid degradation is likely to occur beyond twenty-four hours PM. Further metabolomics studies should consider a longer lapse of time to capture the end point of the purine degradation pathway. Other molecules such as taurine, betaine and *myo*-inositol, modulated in almost every organ, are associated with osmoregulation (Trachtman et al. 1988; Burg and Ferraris 2008).

Niacinamide was also found modulated in several organs in this study. This is consistent with a previous report in the literature that linked it to PM processes in salmons (Shumilina et al. 2015). Other metabolites, such as phenylalanine and tyrosine may result from proteolysis but have also been associated with microbial activity in living organisms (Davila et al. 2013; Neis et al. 2015).

This proof-of-concept work shows the potential of NMR metabolomics in a new field: Thanatometabolomics. Mass-Spectrometry could also be explored as a potential alternative to study PM metabolomic changes. In further investigations, differences between sex and environmental conditions should be considered, which would require a higher number of individuals.

Interestingly, recent studies have suggested that the study of the thanatomicrobiome could be used by forensic microbiologists to estimate the time of death (Javan et al. 2016a, b; Can et al. 2014). This, along with thanotometabolomics and thanatotranscriptomics could provide new forensic tools for the prediction of the time of death.

## Conclusion

To the best of our knowledge, this proof of concept study represents the largest metabolomics characterisation of post-mortem metabolic modulations. Kidney and spleen were the organs that displayed the largest metabolic modulations over the 3 time-points investigated. The reported metabolic perturbations are consistent with anaerobic metabolism and show molecular mechanisms of compensation for the lack of oxygen at the cellular level. Yet, further studies are required to validate the use of these potential biomarkers in humans. These should consider the impact of temperature, gender and environmental conditions in the metabolic fluctuations occurring after death. Integration of thanatometabolomics with other omics’ technologies such as thanatomicrobiology and thanatotranscriptomics would also likely contribute to refine prediction of the time of death.

## Electronic supplementary material

Below is the link to the electronic supplementary material.

Supplementary material 1 (DOCX 19 KB)

Supplementary material 2 (PNG 1116 KB)

Supplementary material 3 (XLSM 16241 KB)

## Data Availability

Data is available in Supplementary material S3.

## Abbreviations

NMR: Nuclear magnetic resonance
TP: Time point
