# Supplementary material for: Thanatometabolomics: introducing NMR-based metabolomics to identify metabolic biomarkers of the time of death
Source: Metabolomics. 2019 Mar 5;15(3):37. doi: 10.1007/s11306-019-1498-1 (PMC6476858; doi:10.1007/s11306-019-1498-1)
Supplement: Supplementary file 1 — Supplementary material 1 (DOCX 19 KB) [file 11306_2019_1498_MOESM1_ESM.docx]

**Table 1.** Metabolic changes occurring in each matrix at different time points.

| **Increased** | **TP1 versus TP2** | | **TP1 versus TP3** | | **TP2 versus TP3** | |
| --- | --- | --- | --- | --- | --- | --- |
|  | **TP1** | **TP2** | **TP1** | **TP3** | **TP2** | **TP3** |
| **Heart** | R^2^Y=0.94, Q^2^Y= 0.60 | | R^2^Y=0.97, Q^2^Y= 0.71 | | R^2^Y=0.96, Q^2^Y= 0.44 | |
|  | Inosine, Creatine, Glucose, Taurine, Carnitine, Choline, Glutamate, Niacinamide, IMP, ATP and Fumarate | Lactate, Glycerol, Malonate, Acetate, Leucine, Valine, Hypoxanthine, Xanthine, Uracil, Phenylalanine and Tyrosine | Inosine, Creatine, Taurine, Choline, Glutamine, Glutamate, Niacinamide, IMP, ATP, adenine, fumarate and uracil. | Glucose, Lactate, Malonate, Acetate, Leucine, Lactate, Valine, Xanthine, Phenylalanine and Tyrosine. | Glucose, Lactate, Malonate, Leucine, Valine, ATP, Xanthine, Phenylalanine and Tyrosine. | Threonine, Creatine, Taurine, Choline, Glutamine, Glutamate, Niacinamide, IMP, Inosine, Adenine, Uracil and Fumarate |
| **Kidney** | R^2^Y=0.95, Q^2^Y= 0.61 | | R^2^Y=0.99, Q^2^Y= 0.87 | | R^2^Y=0.96, Q^2^Y= 0.73 | |
|  | Ascorbate, Inosine, Betaine, Taurine, Choline, Malonate, Glutamate, Niacinamide, UDP-glucose and Uracil. | Glucose, Lactate, Creatine, Acetate, Leucine, Alanine, Valine, Xanthine, Adenine, Phenylalanine, Tyrosine and Fumarate. | Inosine, Betaine, Taurine, Choline, Malonate, Glutamate, Valine, Niacinamide, Adenine, Histidine, Niacinamide, Uracil, Fumarate, UDP-Glucose, Uracil. | Glucose, Lactate, Creatine, Acetate, Leucine, Alanine, Valine, Xanthine, Phenylalanine, Tyrosine. | Inosine, *myo*-inositol, Betaine, Taurine, Malonate, Glutamate, Niacinamide, Inosine, Adenine, Histidine, Niacinamide, Uracil, Fumarate, Inosine, Uracil | Creatine, glucose, Acetate, Leucine, Xanthine, Phenylalanine, Tyrosine and Glucose. |
| **Liver** | R^2^Y=0.84, Q^2^Y= 0.64 | | R^2^Y=0.89, Q^2^Y= 0.65 | | R^2^Y=0.83, Q^2^Y= 0.50 | |
|  | Maltose, Glutamate, Inosine, Histamine, Xanthine, Uracil, Uracil and Maltose | Acetate | Glutathione, Niacinamide, Inosine, Adenine and Maltose. | Creatine, Acetate, Leucine, Valine, Phenylalanine, Tyrosine and Uridine | Glucose, Lipids and Inosine | Maltose, Creatine, Glutamate, Acetate, Leucine, Valine, Phenylalanine, Tyrosine and Uridine. |
| **Spleen** | R^2^Y=0.87, Q^2^Y= 0.68 | | R^2^Y=0.98, Q^2^Y= 0.88 | | R^2^Y=0.97, Q^2^Y= 0.70 | |
|  | Glucose, Ascorbate, Creatine, Taurine, Glutamate, Niacinamide, Inosine, Adenine, Guanosine, Fumarate, UDP glucose and Uridine. | Lactate, Anserine, Acetylcholine, Acetate, Alanine, Valine, Leucine, Formate, Xanthine, Uracil, Phenylalanine, Tyrosine and Histamine. | Ascorbate, O-phophoethanolamine, Glucose, Taurine, Choline, Anserine, Glutamate, Niacinamide, Inosine, Adenine, Uridine, Fumarate, Inosine, UDP-glucose and Uridine. | Lactate, Creatine, Glycerol, Glutamine, Acetylcholine, Acetate, Leucine, Alanine, Valine, Xanthine, Uracil, Phenylalanine and Tyrosine. | Ascorbate, O-phosphoethanolamine, Taurine, Choline, Anserine, Glutathione, Glutamate, Niacinamide, Inosine, Adenine, Uridine, Fumarate and UDP-glucose. | Creatine, Glucose, Acetate, Leucine, Alanine, Valine, Uracil, Phenylalanine and Tyrosine. |
| **Skin** | R^2^Y=0.83, Q^2^Y= 0.31 | | R^2^Y=0.88, Q^2^Y= 0.60 | | R^2^Y=0.80, Q^2^Y= 0.21 | |
|  | Maltose, Glucose, Glycerine, Taurine, Glutamate and Inosine. | Lactate, Acetate, Alanine and Fumarate. | Maltose, Glycerine, Taurine, Inosine. | Lactate, Creatine, Acetate, Alanine, Phenylalanine, Tyrosine, Histamine. | Lactate, Fumarate and Maltose. | Creatine, Phenylalanine, Tyrosine and Histamine. |
| **WAT** | R^2^Y=0.95, Q^2^Y= 0.80 | | R^2^Y=0.80, Q^2^Y= 0.47 | | R^2^Y=0.95, Q^2^Y= 0.83 | |
|  | Lactate, Creatine, Malate, Glutamate, Acetate, Alanine, Leucine, Isoleucine, Valine, Phenylalanine, Tyrosine, Histamine. | Glucose, myo-Inositol, Lipids, Inosine, Glucose, Niacinamide. | Lactate, Myo-Inositol, Taurine, O-phosphoethanolamine, Glutamate. | Creatine and Phenylalanine. | Glucose, myo-Inositol, Taurine, Lipids, Niacinamide, Inosine, Uracil, Fumarate, UDP-glucose, Uridine, Glucose. | Lactate, Creatine, Acetate, Leucine, Isoleucine, Valine, Phenylalanine, Tyrosine, Histamine. |
