# Supplementary figures and images for: Thanatometabolomics: introducing NMR-based metabolomics to identify metabolic biomarkers of the time of death
Source: Metabolomics. 2019 Mar 5;15(3):37. doi: 10.1007/s11306-019-1498-1 (PMC6476858; doi:10.1007/s11306-019-1498-1)

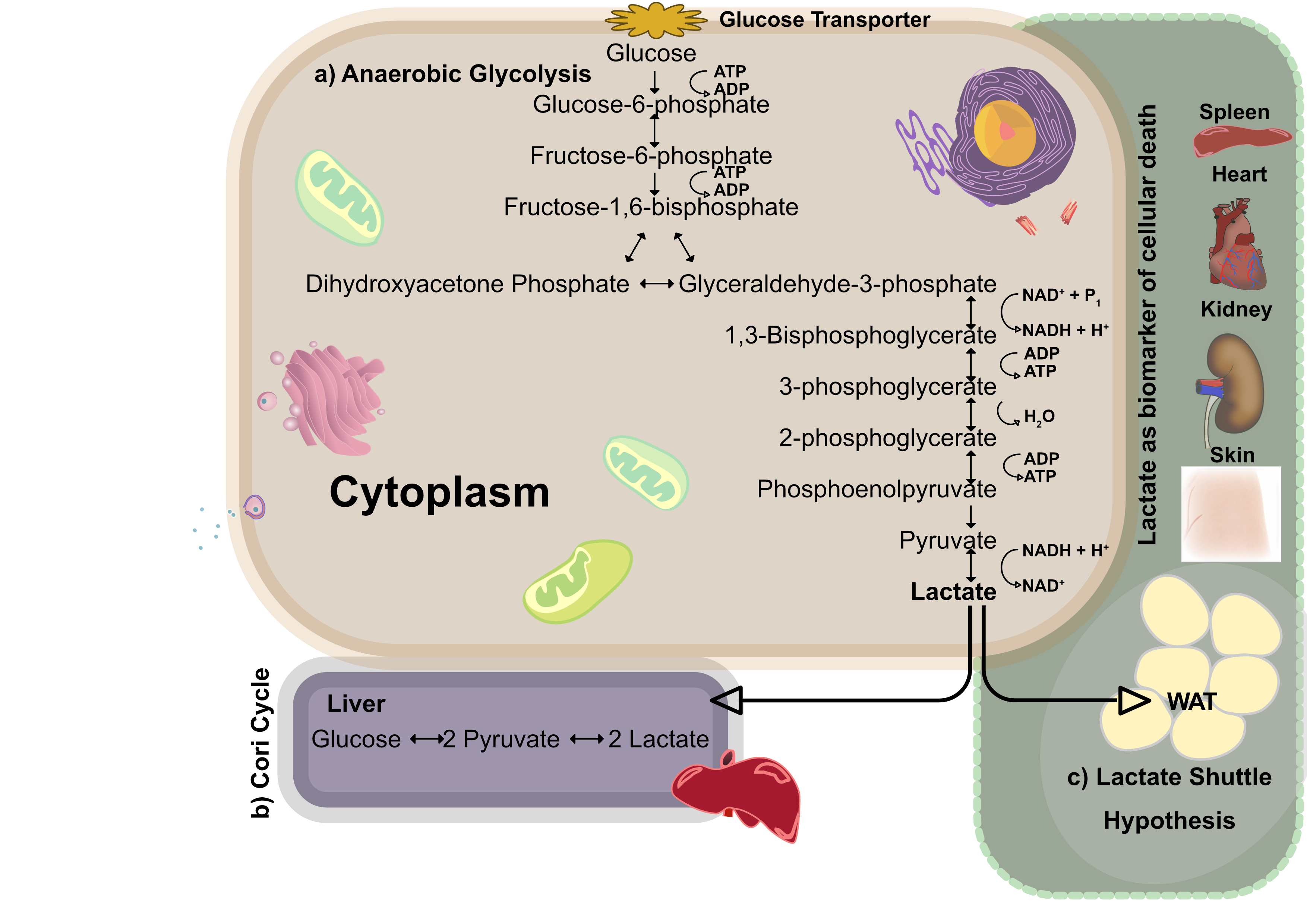

Supplement: Supplementary file 2 — Supplementary material 2 (PNG 1116 KB) [file 11306_2019_1498_MOESM2_ESM.png]
